# Clinical Outcomes of Proximal Metatarsal Closed-Wedge Osteotomy Using Compression Staples and Headless Screw Fixation for Hallux Valgus With Concomitant Lesser Toe Surgeries

**DOI:** 10.7759/cureus.90844

**Published:** 2025-08-23

**Authors:** Asyumaredha Asril Silan, Tomoyuki Nakasa, Yasunari Ikuta, Shingo Kawabata, Dan Moriwaki, Satoru Sakurai, Saori Ishibashi, Nobuo Adachi

**Affiliations:** 1 Orthopedic and Traumatology Division, Muhammad Djamil Central Hospital, Padang, IDN; 2 Department of Artificial Joints and Biomaterials, Graduate School of Biomedical and Health Sciences, Hiroshima University, Hiroshima, JPN; 3 Department of Orthopedic Surgery, Graduate School of Biomedical and Health Sciences, Hiroshima University, Hiroshima, JPN

**Keywords:** compression staple, hallux valgus, lesser toe, osteotomy, screw

## Abstract

Background: Hallux valgus (HV) is a prevalent forefoot deformity that often needs corrective surgery to alleviate pain, improve footwear compatibility, and enhance gait function. Proximal metatarsal osteotomy (PMO) is commonly performed for moderate to severe HV; however, the fixation method for the osteotomy site is still debatable. Additionally, HV correction is frequently accompanied by concomitant lesser toe surgeries, which may lead to increased risks of wound complications and residual symptoms. This study aimed to evaluate the clinical and radiographic outcomes of PMO using a combination of compression staple and screw fixation.

Methodology: A retrospective review of 56 feet in 46 patients (mean age: 65.1 ± 13.6 years) treated with PMO using compression staple and screw fixation was conducted. Clinical outcomes were assessed using the Japanese Society for Surgery of the Foot (JSSF) scale and the Self-Administered Foot Evaluation Questionnaire (SAFE-Q). Radiographic parameters were analyzed preoperatively and at final follow-up.

Results: The mean follow-up period was 18.5 ± 7.4 months. Significant improvements were observed in JSSF scores and all SAFE-Q subscales postoperatively in isolated PMO and multiple lesser toe surgeries. Radiographic analyses demonstrated significant correction of the HV angle, first-second intermetatarsal angle, and first-fifth intermetatarsal angle. No nonunion or major adverse events were recorded, and the recurrence rate was 3.6%.

Conclusions: Postoperative results were good when osteotomy of the metatarsal bone was performed with compression staples and screw fixation. The use of compression staples offers the advantages of persistent compression, reduced invasiveness, and shorter operative time, making it a viable option for HV correction.

## Introduction

Hallux valgus (HV) is the most common forefoot deformity, which affects 23%-36% of the population [1]. To improve symptoms such as pain, difficulty using footwear, and impaired gait, corrective surgery is performed and significantly improves patients' quality of life [2]. Among the various corrective osteotomies, proximal metatarsal osteotomy (PMO) is often more popular for moderate to severe HV deformities because the increased intermetatarsal angle can be better corrected with PMO than with distal and midshaft osteotomies [3]. Several fixation procedures, such as Kirschner wires, screws, and locking plates, have been used to achieve optimal reduction and stable fixation. However, these fixation methods have disadvantages, such as loss of correction, additional soft tissue dissection to place a plate, and skin irritation due to non-precise placement of a plate [4,5]. In addition, HV is often complicated by symptoms of lesser toes, such as dislocations, metatarsalgia, and bunionette, and concomitant surgeries are often performed simultaneously to prevent residual symptoms or transfer metatarsalgia [6]. However, HV corrections with these concomitant surgeries are reported to increase the risk of wound issues such as delayed wound healing or infection due to impaired skin perfusion caused by multiple incisions, poor circulation due to extensive dissection, and residual pain with stiffness, resulting in unsatisfactory postoperative outcomes [7]. Therefore, fixation procedures that easily and firmly fix the osteotomy site without an extensive soft tissue dissection are required.

Recently, compression staples have become widely used in foot and ankle surgeries such as osteotomy and arthrodesis [8,9,10]. Compression staples are capable of persistent compression and can be used in small spaces due to their unique material properties of superelasticity and shape memory [9]. These features allow for easy fixation of the osteotomy site, and in combination with screw fixation, would provide firm fixation in the PMO. In addition, a compression staple can easily fix the osteotomy with a small skin incision in metatarsal shortening osteotomy, which is also advantageous for concomitant surgeries for PMO. We hypothesized that PMO using the combination of a compression staple and a screw fixation would yield good clinical outcomes, even if surgeries on the lesser toes with compression staples or headless screws were performed simultaneously. The purpose of this study was to evaluate the clinical outcomes of PMO using a compression staple and screw fixation, including concomitant surgeries for lesser toes in patients with HV.

## Materials and methods

Participants

Fifty-six feet in 46 patients with HV treated by PMO using a compression staple and screw fixation between April 2021 and December 2023 were retrospectively reviewed. They comprised three men and 43 women, with a mean age of 65.1 ± 13.6 (range, 24-87) years. Ten patients were involved in both feet. Patients who were not followed up for more than one year were excluded. This study was approved by the local ethics committee of our institution, and informed consent was obtained from all participants.

Surgical procedure

**Figure 1:**
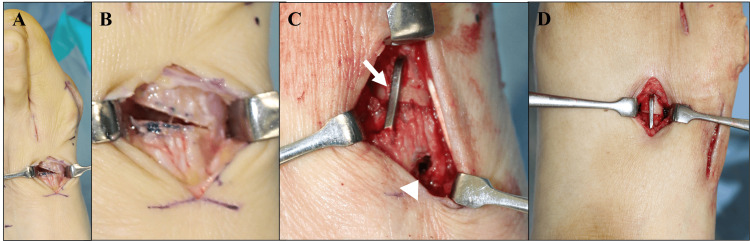
Proximal closed wedge osteotomy (PCWO) and second metatarsal shortening osteotomy. (A, B) After wedge osteotomy at the proximal metatarsal. (C) Osteotomy site of the first metatarsal was fixed using a compression staple (arrow) and headless screw (arrowhead). (D) Fixation using the compression staple at the second metatarsal.

For the correction of HV, proximal closed wedge osteotomy (PCWO) was performed. A dissection of the adductor hallucis tendon from its attachment to the proximal phalanx was performed for the lateral release procedure. Additionally, the lateral metatarsal-sesamoid suspensory ligament and the lateral joint capsule were released. Subsequently, the medial eminence of the first metatarsal head was exposed and removed to preserve the articular surface. A closed wedge osteotomy was performed at the proximal first metatarsal (Figures 1A, 1B). The osteotomy was carefully performed to avoid dorsiflexion at the osteotomy site with a large dorsal cut. The osteotomy angle was determined to make the distal fragment of the first metatarsal parallel to the second metatarsal. The osteotomy site was temporarily fixed using a Kirschner wire with supination of the distal fragment. Subsequently, a compression staple (DynaNite, 15 × 12 mm; Arthrex Inc., Naples, FL) was inserted laterally from the center of the osteotomy side, which persistently provided a compression force at the lateral side of the osteotomy site (Figure 1C). Once fluoroscopy confirmed proper alignment in which the distal fragment of the first metatarsal and the second metatarsal were parallel, the medial side of the osteotomy site received additional fixation with a 3.0 mm headless screw (Wright Medical, Memphis, TN) (Figure 1C). Finally, the medial capsule plication was performed to secure the great toe in its proper alignment. Along with the PMO, additional surgeries, such as shortening or corrective osteotomies, for the lesser toes were performed. For the shortening osteotomy of the second, third, and fourth metatarsals, the osteotomy was performed at the base of each metatarsal in a plane perpendicular to its longitudinal axis. The extent of resection was carefully determined to restore a smooth metatarsal arc, and the corresponding bone segment was excised. The osteotomy surfaces were anatomically aligned and brought into contact, and stable fixation was achieved using a compression staple (DynaNite, 15 × 12 mm or 13 × 10 mm) (Figure 1D). For patients with bunionette, an oblique osteotomy at the fifth metatarsal shaft with fixation using a 2.5 mm headless screw (Wright Medical) was performed [11]. Weight-bearing was permitted one week after surgery under a reverse camber orthosis (Nakamura Brace, Shimane, Japan). Five weeks after surgery, the reverse camber orthosis was changed to an insole with a metatarsal pad and arch support. Three months after surgery, the insole was removed, and unrestricted activities were allowed.

Clinical evaluation

Clinical outcomes were assessed using the Japanese Society for Surgery of the Foot (JSSF) Hallux metatarsophalangeal-interphalangeal scale preoperatively and postoperatively at the final follow-up [12,13]. Patient-reported outcomes were evaluated using the Self-Administered Foot Evaluation Questionnaire (SAFE-Q), comprising five subscales: (1) pain and pain-related; (2) physical functioning; (3) social functioning; (4) shoe-related; and (5) general health and well-being preoperatively and postoperatively at the final follow-up [14,15,16].

Radiographic evaluation

Using preoperative weight-bearing anteroposterior and lateral radiographs, the following parameters were measured preoperatively and at the final follow-up: hallux valgus angle (HVA), first-second intermetatarsal angle (M1M2 angle), first-fifth intermetatarsal angle (M1M5 angle), Meary’s angle, and calcaneal pitch angle. The recurrence of the HV was defined as 20° ≤ HVA according to a previous report [17].

Statistical analysis

Paired t-tests were used to compare the JSSF scale, SAFE-Q, and radiographic parameters before and after surgery. Multiple comparisons were performed using the Kruskal-Wallis test. Statistical significance was set at *P* < 0.05. Two orthopedic surgeons performed measurements of the radiographic parameters twice by each observer, with a duration of at least 30 days between the measurements and their previous measurements. Intra- and inter-observer reliabilities were analyzed using intraclass correlation coefficients (ICCs). The ICC values were interpreted as follows: <0.40, poor agreement; 0.40 to 0.75, fair to good agreement; and >0.75, excellent agreement.

## Results

The mean follow-up period was 18.5 ± 7.4 months (range: 12-36). The JSSF scale and all subscales of the SAFE-Q significantly improved from preoperative to the final follow-up. In the radiographic analyses, HVA, M1M2 angle, and M1M5 angle were significantly improved from preoperative to final follow-up; however, there were no significant differences in Meary’s angle and calcaneal pitch angle between preoperative and final follow-up (Table 1). There were no cases of nonunion, breakage of the staple, or adverse events such as wound trouble. Recurrence of HV deformity occurred in two feet (3.6%), both of which were cases with poor bone quality due to rheumatoid arthritis. One foot showed transfer metatarsalgia at the second MTP joint. There were no feet to require revision surgery.

**Table 1:** Summary of clinical outcomes. JSSF, Japanese Society for the Surgery of the Foot; SAFE-Q, Self-Administered Foot Evaluation Questionnaire; HVA, hallux valgus angle; M1M2, first-second intermetatarsal; M1M5, first-fifth intermetatarsal; CP, calcaneal pitch

	Preoperative	Final follow-up	*P*-value
JSSF scale (points)	51.5 ± 5.5 (37-57)	88.4 ± 6.9 (75-100)	<0.001
SAFE-Q			
Pain and pain-related	59.7 ± 18.7 (16.7-97.7)	93.0 ± 9.6 (67.2-100)	<0.001
Physical functioning	71.0 ± 17.3 (20.5-100)	95.7 ± 5.1 (81.8-100)	<0.001
Social functioning	74.0 ± 22.3 (16.7-100)	99.3 ± 2.7 (83.3-100)	<0.001
Shoe-related	37.3 ± 19.3 (0-93.3)	84.8 ± 12.7 (58.3-100)	<0.001
General health and well-being	69.6 ± 20.8 (5-100)	98.2 ± 4.7 (80-100)	<0.001
HVA (°)	43.6 ± 8.3 (26-57)	9.7 ± 5.9 (0-25)	<0.001
M1M2 angle (°)	19.9 ± 4.2 (12.5-28.7)	8.5 ± 2.7 (4.2-15.5)	<0.001
M1M5 angle (°)	38.9 ± 5.5 (28.5-57.2)	24.1 ± 5.0 (15.8-37.7)	<0.001
Meary's angle (°)	6.0 ± 5.3 (-7 to 19)	7.4 ± 5.4 (-1 to 17)	0.085
CP angle (°)	15.2 ± 4.0 (6.7-23.9)	15.0 ± 3.9 (7.8-24)	0.298

**Figure 2:**
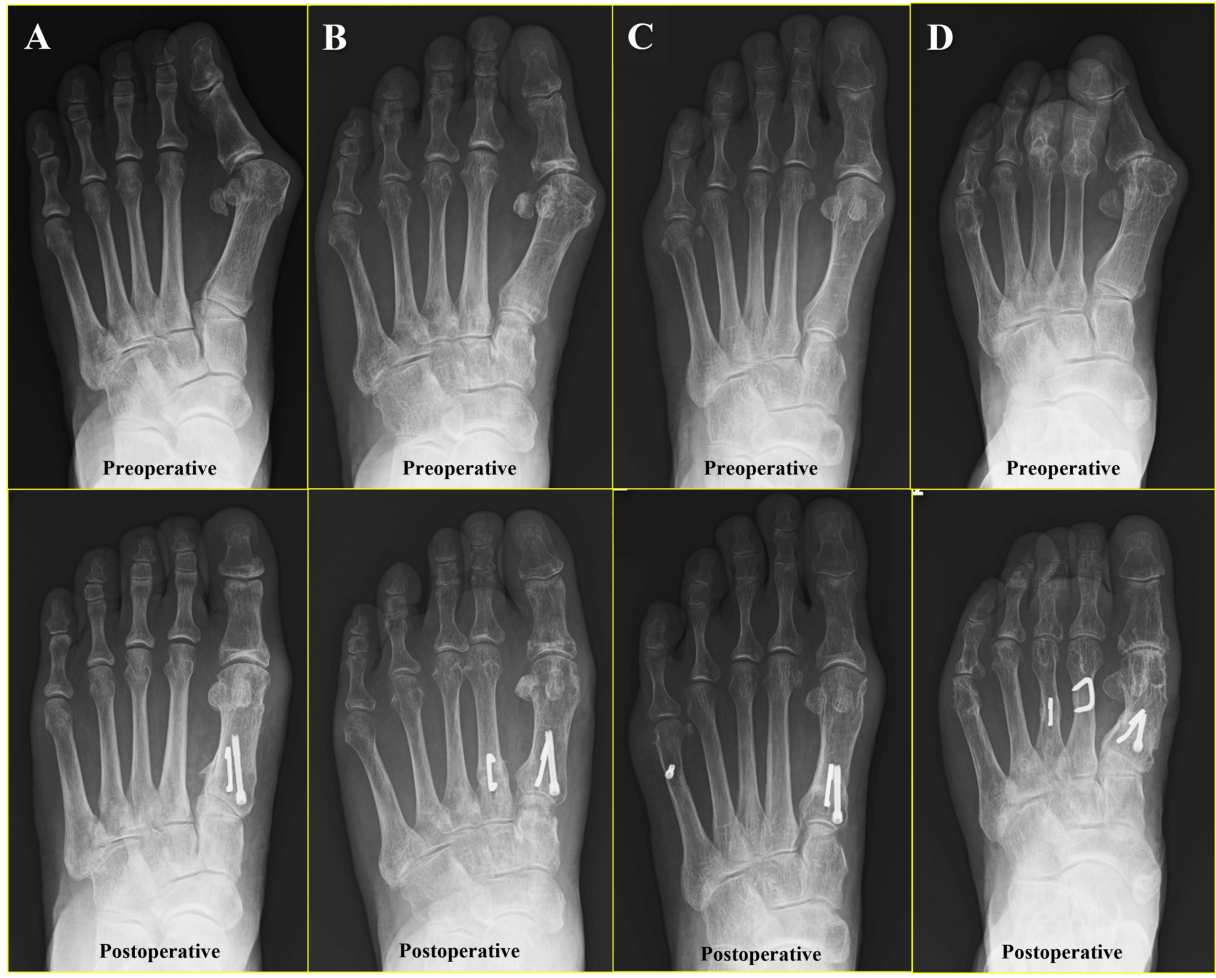
Representative plain radiographs of each procedure. (A) Isolated PCWO. (B) PCWO with shortening of the second metatarsal. (C) PCWO with oblique diaphyseal osteotomy. (D) PCWO with multiple surgeries for lesser toes.

Isolated PCWO was performed for 9 feet (16.1%), PCWO with the second metatarsal shortening osteotomy for 20 feet (35.7%), PCWO with oblique diaphyseal osteotomy of the fifth metatarsal for 13 feet (23.2%), and PCWO with multiple surgeries, two or more osteotomies of the second to fifth metatarsals, for 14 feet (25%) (Figure 2). The JSSF scale and all subscales of the SAFE-Q significantly improved from preoperative to the final follow-up in all groups. In the radiographic analysis, the HVA, M1M2 angle, and M1M5 angle significantly improved from the preoperative assessment to the final follow-up in all groups. However, there were no significant differences in Meary’s angle or the calcaneal pitch angle between the preoperative assessment and the final follow-up. Preoperatively, HVA was significantly smaller in the PCWO with 2MSO than in the PCWO with ODO (*P *< 0.05). In the SAFE-Q, the subscales for pain and pain-related issues, physical function, and shoe-related factors were lower in the PCWO with multiple surgeries group compared to the PCWO with 2MSO group (*P* < 0.05) (Table 2). Postoperatively, the JSSF scale was higher in the isolated PCWO than in the other three groups (*P* < 0.05). The HVA and M1M2 angles were significantly smaller in the PCWO with ODO than in the PCWO with multiple surgeries (*P* < 0.05) (Table 3). 

**Table 2:** Preoperative parameters. ^a^Significant difference between PCWO + 2nd SO and PCWO + multiple surgeries (*P* < 0.05). ^b^Significant difference between PCWO + 2nd SO and PCWO + ODO (*P* < 0.05). SO, shortening osteotomy; ODO, oblique diaphyseal osteotomy; HVA, hallux valgus angle; M1M2, first-second intermetatarsal; M1M5, first-fifth intermetatarsal; CP, calcaneal pitch; PCWO, proximal closed wedge osteotomy

	PCWO (*n* = 9)	PCWO + 2nd SO (*n *= 20)	PCWO + ODO (*n* = 13)	PCWO + multiple surgeries (*n* = 14)
JSSF scale (points)	53.6±4.5 (47-57)	51.3 ± 5.3 (37-57)	52.6 ± 4.7 (47-57)	49.5 ± 6.9 (37-57)
SAFE-Q				
Pain and pain-related	61.5 ± 18.5 (16.7-87.7)	66.6 ± 22.4 (21.7-97.2)^a^	54.4 ± 16.9 (21.7-76.7)	53.6 ± 10.4 (29.4-68.9)
Physical functioning	70.1 ± 10.2 (54.5-86.4)	77.8 ± 18.3 (40.9-100)^a^	68.0 ± 17.4 (40.9-95.5)	64.6 ± 17.7 (20.5-88.6)
Social functioning	76.4 ± 16.8 (45.8-100)	77.7 ± 23.6 (25-100)	71.2 ± 25.1 (25.0-100)	69.6 ± 22.1 (16.7-100)
Shoe-related	42.8 ± 27.2 (16.7-93.3)	41.9 ± 18.3 (0-66.7)^a^	34.4 ± 11.1 (16.7-50)	29.8 ± 19.8 (0-66.7)
General health and well-being	71.1 ± 31.8 (5-100)	72.0 ± 18.5 (40-100)	70.4 ± 19.7 (40-100)	64.6 ± 17.6 (20-85)
HVA (°)	45.9 ± 10.2 (32-59)	45.4 ± 7.6 (26-56)^b^	39.2 ± 7.3 (28-47.7)	43.7 ± 8.0 (34-57)
M1M2 angle (°)	20.1 ± 3.7 (14-24.5)	20.9 ± 4.7 (14.1-28.7)	19.5 ± 4.1 (14.6-27.5)	18.6 ± 4.1 (12.5-28)
M1M5 angle (°)	36.8 ± 5.9 (28.5-46.1)	39.1 ± 4.9 (29.3-47.2)	41.2 ± 4.4 (34.6-51.0)	38.0 ± 6.8 (31.7-57.2)
Meary's angle (°)	6.8 ± 7.5 (0-19)	6.5 ± 4.7 (0-17)	3.7 ± 2.9 (0-8.1)	7.1 ± 6.1 (-7-16)
CP angle (°)	15.5 ± 4.9 (6.7-21.3)	14.7 ± 3.0 (8.6-22.6)	16.1 ± 5.4 (7.4-23.3)	15.2 ± 3.5 (8.9-23.9)

**Table 3:** Postoperative parameters. ^a^Significant difference between isolated PCWO and PCWO + 2nd SO (*P *< 0.05). ^b^Significant difference between PCWO + ODO and PCWO + multiple surgeries (*P *< 0.05). ^c^Significant difference between isolated PCWO and PCWO + 2nd SO (*P *< 0.05). SO, shortening osteotomy; ODO, oblique diaphyseal osteotomy; HVA, hallux valgus angle; M1M2, first-second intermetatarsal; M1M5, first-fifth intermetatarsal; CP, calcaneal pitch; PCWO, proximal closed wedge osteotomy

	PCWO (*n *= 9)	PCWO + 2nd SO (*n *= 20)	PCWO + ODO (*n *= 13)	PCWO + multiple surgeries (*n *= 14)
JSSF scale (points)	93.9 ± 6.0 (85-100)^a^	87.2 ± 6.2 (77-100)	90.8 ± 5.5 (85-100)^b^	84.2 ± 6.7 (75-100)^c^
SAFE-Q				
Pain and pain-related	97.3 ± 2.9 (92.2-100)	95.0 ± 7.8 (78.3-100)	90.0 ± 12.7 (67.2-100)	90.2 ± 10.3 (70.6-100)
Physical functioning	98.0 ± 3.1 (90.9-100)	95.7 ± 4.4 (83.6-100)	94.9 ± 6.0 (81.8-100)	95.2 ± 6.1 (81.8-100)
Social functioning	100	98.5 ± 4.1 (83.3-100)	100	99.4 ± 2.2 (91.7-100)
Shoe-related	83.0 ± 18.3 (58.3-100)	85.0 ± 11.3 (58.3-100)	85.3 ± 10.8 (66.7-100)	85.1 ± 13.1 (66.7-100)
General health and well-being	99.4 ± 1.7 (95-100)	99.3 ± 1.8 (95-100)	100	94.3 ± 8.1 (80-1009
HVA (°)	12.1 ± 5.7 (6-25)	9.9 ± 5.2 (0-18)	6.5 ± 5.0 (0-18)^b^	10.8 ± 7.2 (0-21)
M1M2 angle (°)	7.4 ± 1.8 (5-10)	8.9 ± 1.8 (5.8-12.3)	7.5 ± 2.3 (4.2-12)^b^	9.4 ± 4.0 (5-17)^c^
M1M5 angle (°)	25.0 ± 6.8 (17.5-37.7)	25.6 ± 4.1 (17.9-29.6)	22.0 ± 4.1 (16.9-31.3)	23.3 ± 5.4 (16.6-34.6)
Meary's angle (°)	8.1 ± 6.8 (-1-17)	7.6 ± 5.1 (1-21)	4.3 ± 3.2 (0-9)	9.6 ± 5.7 (0-23)
CP angle (°)	14.6 ± 6.0 (9-24)	13.3 ± 3.1 (9-19)	15.0 ± 4.5 (7.8-21.6)	12.5 ± 2.4 (8.9-16.4)

Both the intraobserver (0.921; 95% confidence interval (CI), 0.866-0.954) and interobserver (0.853; 95% CI, 0.758-0.913) ICCs for the measurement of radiographic parameters were excellent.

## Discussion

This study showed that PCWO using a compression staple and a headless screw achieved good clinical outcomes, even with concomitant surgeries on the lesser toes. The use of compression staples has the advantages of providing persistent pressure to the osteotomy site to achieve a corrective position in PCWO and easily fixing the osteotomy site in metatarsal shortening. Compression staple and screw fixation of the osteotomy site is suitable for corrective surgery of forefoot deformity. 

Stable fixation of the osteotomy site is crucial for successful outcomes in PMO, although it is well-established. Locking plates have emerged as a superior option to K-wires or screws for fixation in PMO. It is reported that the dorsal plate is biomechanically more stable than a single screw in the proximal crescentic osteotomy [18]. Pauli et al. reported that proximal crescentic osteotomy using a head-locking X-plate yielded satisfactory and reproducible results in terms of stability, clinical outcomes, bone healing, and patient satisfaction [4]. Although these reports indicate that fixation with a locking plate can solve the problem of stabilization at the osteotomy site during PMO, several problems remain when using a locking plate. It is reported that the recurrence of HV after surgery is strongly related to patient satisfaction [19], and the recurrence rates after PMO range from 4% to 25% [20]. Park et al. reported that fixation using a plate and screws has a greater risk of HV recurrence than fixation using Kirschner wires in PMO [5]. In their report, the mean intermetatarsal angle and first and second metatarsal distance were larger in the plate group on the immediate postoperative radiograph, indicating that correction loss may occur during plate fixation [5]. On the other hand, fixation using a compression staple applied to the lateral side of the osteotomy site does not theoretically induce loss of the corrective angle at the osteotomy site by a persistent compression force. In addition, placement of a locking plate requires large soft tissue dissection, with difficulties in precisely placing it on the irregular bony surface, which may induce complications such as skin irritation [5]. Compared to a locking plate, compression staples are small in size and have a low profile, allowing insertion in any location, regardless of metatarsal bone morphology. Their small size and low-profile design necessitate less soft tissue dissection and lead to fewer implant-related complications compared to the bulkier locking plate.

Recently, compression staples have become widely used in foot surgeries. Horner et al. reported that compression staples provided robust and stable fixation for Akin osteotomies and had low complication rates and high healing rates [21]. Dock et al. reported that 89.7% of the fusion rate in tarsometatarsal joint arthrodesis using compression staples and patient satisfaction was consistent with that of other fixation methods [22]. The increased use of compression staples may be due to their advantages over other fixation procedures. Compression staples can provide continuous compression force owing to the unique properties of nitinol, which is composed of nickel and titanium. Several biomechanical studies have demonstrated its ability to maintain the compression force across joints during arthrodesis, suggesting that this ability is also maintained in osteotomies [8,22]. A previous report showed that a compression staple placed laterally in the ankle joint could help achieve proper alignment in arthrodesis for a varus-type ankle osteoarthritis [9]. In addition, compression staples allow compression throughout the resorptive phase of fusion, which occurs when screws typically lose their compression and stability [23]. Compression staples can maximize the total contact area at the osteotomy site without multiple crossings of the fusion surface using screws [8,24]. In our procedure, fixation using a headless screw was added to a single compression staple because one compression staple alone has weak resistance to rotational and shear forces. A previous report demonstrated that double staple fixation had the most consistent bending stiffness in all planes compared to single staple and plate fixation [8]. In PMO, the dorsal elevation of the metatarsal during bone healing and the metatarsal shortening are the major concerns [3]. Therefore, a headless screw was inserted in a proximal dorsal to the distal plantar direction to prevent dorsal elevation of the distal fragment induced by the compression staple in our procedure.

HV is frequently associated with the problems of lesser toe deformity, such as bunionette, hammertoes, and plantar callosity [25]. It is reported that metatarsalgia under the second and/or third metatarsal heads occurs in moderate to severe HV due to an increase in the relative length of the second and/or third metatarsals [26]. Therefore, lesser metatarsal shortening osteotomy and bunionette correction are often performed simultaneously, with good clinical outcomes [3,27]. In our series, patients who underwent multiple surgeries had more complaints about pain, physical function, and shoes preoperatively. For these patients, good clinical outcomes were obtained by performing surgeries on symptomatic areas simultaneously. Low-invasive and less time-consuming procedures may be necessary for successful outcomes of multiple surgeries. Previous reports demonstrated that compression staples are easy to insert, which results in reducing the operation time [10,28]. The use of compression staples to fix osteotomy sites in forefoot deformity correction can be useful for obtaining good clinical outcomes, even in multiple surgeries for lesser toes combined with HV correction. 

This study has several limitations. First, the number of patients was small, and the follow-up period was short because this surgical procedure had started as soon as the compression staples became commercially available. Therefore, the number of cases for each procedure is small, and sufficient analysis has not been performed. Second, this study retrospectively analyzed clinical outcomes using compression staple fixation without comparing them to other fixation methods, such as a locking plate. In addition, the difference in stability between using compression staples alone and using them in combination with screws was not evaluated. To demonstrate the effectiveness of the combined use of compression staples and screws, biomechanical testing using cadaveric specimens would be necessary. Prospective randomized studies compared to other methods in large populations will be required for longer follow-ups. Although various limitations exist, the favorable outcomes observed in this study suggest that it could serve as one of the surgical options for HV, for which multiple procedures are available.

## Conclusions

This study demonstrated good clinical outcomes using compression staple and screw fixation at the osteotomy site in PCWO, even when combined with multiple lesser toe surgeries. A compression staple has the advantage of providing persistent compression force and an easy procedure with less invasive and reduced operation time, and these advantages make it suitable for HV surgeries, including for lesser toes.
